# Record of a Case of Glanders in Man, with History of Infection from a Horse, Together with a Clinical and Post Mortem Record of the Case in the Suspected Animal

**Published:** 1893-02

**Authors:** A. W. Clement

**Affiliations:** Baltimore, Md.


### THE JOURNAL
OF
### COMPARATIVE MEDICINE AND VETERINARY ARCHIVES.

Vol. XIV. FEBRUARY, 1893. No. 2.

### Record of a Case of Glanders in Man with History of Infection from a Horse, Together with a Clinical and Post-Mortem Record of the Case in the Suspected Animal.

By A. W. Clement, V.S.

The death of a man from glanders in this city, in November last, created considerable excitement in the Health Department, and is of such scientific interest, that I venture to send the records to the Journal.

By the kindness of Dr. McShane, Health Commissioner for the city of Baltimore, I am enabled to transmit the record of the autopsy as performed by Dr. N. G. Kierle, the Medical Examiner to the Board of Health. It is as follows:

"Jno. Hartlove, male, white, 34 years of age, died on the 5th day of November, 1892, from Farcy Glanders. Nutrition medium; rigor mortis marked. Height of body, 5 feet 8 inches; weight, 150 lbs. Sub-pleural ecchymoses. Kidneys enlarged, 'cloudy swelling.' Nares swollen, scabbed, ulcerated. Papules sparsely scattered over body; some papules vesicular, collapsed at apices. Slough on inside of left elbow, six inches by three inches in area. Large abscess on inside of left upper arm. Ecchymotic maculæ on inside of right arm.

"Dr. Simon Flexnor, associate in Pathology at the Johns Hopkins Hospital, has kindly furnished me with the following record:

"On November 5th, '92, through the courtesy and by request of Dr. McShane, health officer of the city of Baltimore, I was enabled to see the patient who was supposed to be suffering from acute glanders and to make cultures from the skin abscesses. The clinical features of the case I am unable to give as I saw him only on this occasion, until after his death, which occurred later in the same day in which I made cultures, but through the courtesy of Dr. Kierle, the pathologist of the City Hospital, I was permitted to be present at the autopsy and again make cultures, this time from the viscera.

" The first set of cultures was made from the pus evacuated from a superficial abscess beneath the skin, which had not opened and which was incised with a knife sterilized immediately before in the flame of an ordinary gas burner, no other means being at hand. A platinum loop, sterlized in a similar manner, was introduced into the abscess and a quantity of very stringy and sticky pus transfered to several gelatine beef-broth tubes brought for the purpose. Immediately on reaching the laboratory Petri's plate cultures were made in Guarnieré's culture medium (agar-gelatine beef broth) from the pus, and these were placed in the thermostat at 37° C.

"From another portion of the pus, cover-slip preparations were made and stained in various aniline dyes, gentian-violet, methylene-blue and carbol-fuchsin, mounted in the usual way in balsam and examined microscopically. The microscopical examination of these cover-slips revealed a considerable number of bacilli which corresponded in size and staining properties with the glanders bacillus as described by Lœffler and Schutz, so that it is needless to repeat the description here. A portion of the pus removed in these operations was reserved for future use.

"On November 6th the Petri's plates contained a very large number of minute colonies; indeed, the number was so great that there was no opportunity for individual colonies to grow to maturity. Although not mentioned before, two agar plants were made from the pus at the same time the plates were made, and these, too, showed a growth on the surface. Cover-slip preparations from the cultures showed bacilli possessing the same general character as those found in the pus originally. Two guinea pigs were inoculated on November 8th, one with a small quantity of the pus which remained and the other from an agar culture. The inoculations were made into the subcutaneous tissues of the side, high up, near the vertebral column, about midway of the body. On November 16th, hence 8 days after the inoculations, in both cases an ulcer formed corresponding with the inoculations. The following is the note made at the time: 'The seat of inoculation has appeared for several days red and swollen. To-day there is a well-marked ulceration with elevated edges, the whole about the size of a silver half dollar.'

"On November 19th one of the guinea pigs was found dead early in the morning; the other, at this time, was noted to be in a weak and evidently dying condition. It died at 8 in the evening. The autopsies of these animals were alike in all essential respects. The bacilli were obtained from the seat of inoculation and from the internal organs, such as the liver, spleen and lungs, in which there were numerous white foci, the so-called glanders nodules. Bacilli were also found, although only with difficulty, in sections of the liver of one of the animals. The testicles showed the classical lesion. The skin of the scrotum was tense, the hair had come away in part, and the testicles beneath were evidently much swollen. On section, in the substance of the organ, were large, yellow, necrotic and purulent (?) areas. The material forming these necrotic areas contained the glanders bacilli. A third guinea pig, inoculated with some of the necrotic material of the testicle of the second, died in 23 days. This animal was a female, and in addition to the lesions in the viscera, described in the first two, it had abscesses in the joints of the posterior extremities.

"Cultures made from the viscera of the man dead of glanders did not contain any organisms.

"Some time later (date not recorded) a small quantity of mucus and extraneous matter was sent to the laboratory, with the statement that it was the nasal secretion of the horse supposed to have been the agent of infection in the man. Roll Esmarch cultures made from it did not yield the glanders bacilli, so that a guinea pig was inoculated with the fluid at the bottom of the culture tube containing a variety of organisms. This animal died after about four weeks of glanders.

" The horse was killed, and an autopsy made by Dr. Clement."

I have not been able as yet to obtain a detailed clinical record of Hartlove's case, but in general it is as follows: He was employed as a nurse in a car-stable, and also took care of a horse belonging to his brother. He had a habit of biting his finger nails until the skin would bleed. One day he noticed a swelling on the end of his little finger, which grew rapidly worse and became very painful. An abscess soon formed in the axilla. This was followed by the appearance of sores on other parts of the body, swelling of the nares, hyperæsthesia, and semi-unconsciousness. The attending physician thought that he had a case of septicæmia to deal with, but in consultation the diagnosis of glanders was made. The patient himself was not able to give any intelligent history of his case, and it was not at that time known by the physicians that he had been attending a horse which presented symptoms of glanders.

After the death of Hartlove, an investigation by the health authorities fastened suspicion on the horse that belonged to the deceased's brother, and which had been taken care of by the deceased. On November 15th, Dr. Ward, the State Veterinarian, was called in. Upon investigation, he found that the horse in question belonged to the deceased's brother, who had bought him some six weeks previous. After the horse had been in his (Hartlove's) possession for about a week he noticed that it was running at the nose. The animal was immediately placed in quarantine.

On the following day I saw the horse with the State Veterinarian. The animal presented the following symptoms: Hair somewhat dull, animal in good spirits, well nourished ; slight discharge from both nostrils, but a little more profuse on the right side ; mucous membrane of nostrils normal, so far as could be seen in the bright sunlight ; sub-maxillary glands slightly enlarged, about the size of hickory nuts, hard, but not adherent to the bone ; no enlargement of subcutaneous lymphatic glands could be made out, no farcy buds to be seen, pulse 45, temperature 100 degrees. The case was considered very suspicious, but a positive diagnosis of glanders could not be made.

Dr. Ward obtained some mallein from the Department of Agriculture in Washington, and from the 19th to the 23d, took the animal's temperature preparatory to inoculation. The following is a table of temperature on the different days :

| | | | |
|---|---|---|---|
| Nov. | 19th, | 4.30 P. M | 101 |
| " | 20th, | " | 99.4 |
| " | 21st, | " | 99.3 |
| " | 22d, | " | 99.4 |
| " | 23d, | 12.30 P. M | 100.2 |

Immediately after taking the temperature on the 23d, Dr. Ward inoculated 1 ccm. of the mallein solution under the skin of the neck. At 5 P. M. on the same day the temperature was 100.2.

On the 24th, at 8.45 A. M., 104 degrees, at 11.40 A. M., 102.4, at 4.30 P. M., 102.3.

On the 25th, at 8.45 A. M., 102.1, at 4.30 P. M., 101.4.

From the 25th to the 29th, when the animal was killed, the temperature ranged from 101.4 degrees to 102.2 degrees.

At the request of Dr. Ward, the State Veterinarian, and of Dr. McShane, the Health Commissioner, I made an autopsy upon the animal on the 29th of November.

RECORD OF AUTOPSY.

Subject, dark brown mare of about 1,000 lbs. weight. Body well nourished, no abrasions of the skin, no subcutaneous nodules to be felt, save the submaxillary glands. On removal of the skin the sub-maxillary glands were found to be indurated on section, but presented no necrotic foci. Axillary and inguinal glands normal. On section of the head, the nasal mucous membrane presented a number of cicatrices, and a number of nodules the size of a poppy seed imbedded in the mucous membrane. Just beneath the pleura and in the substance of the lungs were several similar nodules. There were no cicatrices or ulcers in the larynx or trachea. Other organs normal. There were no guinea pigs in the laboratory at the time, nor could any be obtained, hence inoculation with the nasal mucous membrane was not practiced.

Mallein is being prepared, under the direction of Professor Welch, from cultures obtained through guinea pigs from the man who died, and it is thought that an opportunity may be offered to test the material upon some horses which have been exposed to the disease.

BALTIMORE, Md., January 14th, 1893.

NOTE.—Dr. Ward inspected all of the horses in the stable where Hartlove was employed, but could discover no signs of glanders in any of them.

